# Characterization of a Marine Bacterium Passing through a 0.1-μm Pore-sized Filter

**DOI:** 10.1264/jsme2.ME24014

**Published:** 2025-03-08

**Authors:** Haruo Yamaguchi, Kazumasa Yamada

**Affiliations:** 1 Faculty of Agriculture and Marine Science, Kochi University, Monobe-Otsu, Nankoku, Kochi 783–8502, Japan; 2 Faculty of Marine Science and Technology, Fukui Prefectural University, Gakuen-cho, Obama, Fukui 917–0003, Japan

**Keywords:** bacteria, filters, *Saccharospirillum*, slender, 0.1‍ ‍μm

## Abstract

The present study aimed to isolate and characterize a marine bacterium capable of passing through a 0.1-μm pore-sized filter (0.1-μm filter). Sediment suspension samples were filtered through 0.1-μm filters, inoculated into sterile media, and incubated. Isolated SspURN76 belonged to *Saccharospirillum*, according to 16S rRNA gene sequencing, and showed a very slender shape. The minimum cell size of SspURN76 was 0.09×3.2‍ ‍μm. These morphological features of SspURN76 were likely responsible for its passage through 0.1-μm filters. Based on the results obtained herein, marine bacteria may be present in 0.1-μm filtered fractions.

Marine bacteria play central roles in global biological and chemical cycles. Filters with pore sizes ≤0.45‍ ‍μm have been used to collect and/or remove bacterial cells in seawater through filtration procedures (MacDonell and Hood, 1982; Hoff, 1993; Kirchman, 2012). However, not all bacterial cells are trapped by these filters (Duda *et al.*, 2012; Nakai *et al.*, 2013; Obayashi and Suzuki, 2019).

Bacteria have a number of morphological features that contribute to their passage through filters with micropores. MacDonell and Hood (1982) reported that some bacterial populations passed through filters with a pore size of 0.2‍ ‍μm. Nakai *et al.* (2013) filtered coastal waters though 0.2-μm filters and then isolated many species of *Saccharospirillum*, *Reinekea*, and other genera from the filtrates. These small and filterable bacteria show great diversity, as summarized by Ghuneim *et al.* (2018) and Nakai (2020). However, to the best of our knowledge, there is no evidence of marine bacterial isolates passing through not only 0.2-μm filters, but also 0.1-μm pore-sized filters (0.1-μm filters).

In a freshwater ecosystem, Wang *et al.* (2007) found that approximately 0.2% of freshwater bacterial cells were capable of passing through 0.1-μm filters (Fig. S1). *Hylemonella gracilis* (*Betaproteobacteria*) was isolated from 0.1-μm filtrates (Wang *et al.*, 2008). The cells of this isolate had the smallest width (0.2‍ ‍μm), a slender shape, and passed through a 0.1-μm filter (Wang *et al.*, 2008).

In consideration of these issues and marine bacterial diversity, we aimed to confirm the hypothesis that some marine bacterial populations pass through 0.1-μm filters. The present study isolated and characterized a marine bacterium that passes through not only 0.2-μm filters, but also 0.1-μm filters and discussed its morphological features.

Sampling was conducted on the coast of Tosa Bay in Kochi Prefecture, Japan. On July 13, 2020, sediments were collected from the station (33°26′09.6″N, 133°27′36.0″E) using an Ekman–Birge-type bottom sampler (RIGO). The surface sediment (0–2‍ ‍cm) was sampled using a dispensing spoon and transferred into a plastic container. The sample was stored in the dark at –25°C prior to isolation.

Some of the sediment sample was suspended in autoclaved seawater and centrifuged for 1‍ ‍min using a manual centrifuge 1011 (Hettich) with a swing-out rotor 1025 (Hettich). The resultant supernatant was used for bacterial isolation. Cultures of *Karenia papilionacea* (*Dinophyceae*) KpNOM1H (Yamaguchi *et al.*, 2016b) were also used. Preliminary to the present study, we found bacterial growth in a 0.2-μm filtered sample of the culture. These samples were respectively filtered through a 0.2-μm filter (Minisart NML; Sartorius) and then through a 0.1-μm filter (Milex-VV; Merck Millipore). Syringe top-type filters were used in the filtration process. The membrane materials of the 0.2-μm and 0.1-μm filters were cellulose acetate and polyvinylidene fluoride (PVDF), respectively.

Filtrate samples originally obtained from coastal sediments were inoculated into axenic clonal cultures of the diatom, *Chaetoceros* sp. (unidentified). Cultures were maintained in SWM-3 (Chen *et al.*, 1969) with 2 nM Se (Imai *et al.*, 1996). Culturing was conducted at 20°C with 100–120‍ ‍μmol photons m^–2^ s^–1^ (cool white fluorescence) on a 12:12‍ ‍h light:dark cycle. Cell suspensions were filtered as described above.

Serially diluted (10^–1^–10^–11^) samples of the 0.2-μm and 0.1-μm filtrates were prepared with autoclaved seawater. Samples were then inoculated into test liquid media, which contained 0.5‍ ‍g‍ ‍L^–1^ tryptone (Nacalai Tesque) and 0.05‍ ‍g‍ ‍L^–1^ dried yeast extract (Nacalai Tesque). After an incubation at 20°C in the dark, growing bacterial samples were spread onto black-stained test agar media containing 2% (w/v) black color powder (Kyoritsu foods). Solid cultures were then incubated in the dark at 20°C. According to the streak plating method, a bacterial colony was picked, inoculated into the test liquid medium, and then established as the pure culture isolate.

The present experiments investigated the sequence of the 16S rRNA gene region. Cells of the strains were harvested by centrifugation at 8,200×*g* for 10‍ ‍min. Genomic DNA extraction, PCR, and sequencing procedures were performed by Macrogen Japan Corp. Genomic DNA was extracted using PrepMan Ultra Sample Preparation Reagent (Thermo Fisher Scientific). The target region of DNA was amplified using PrimeSTAR HS DNA polymerase (TaKaRa) with 50‍ ‍μM of oligonucleotide primer sets: 27F (5′-AGA GTT TGA TCM TGG CTC AG-3′) and 1492R (5′-TAC GGY TAC CTT GTT ACG ACT T-3′). PCR mixtures were prepared according to the manufacturer’s instructions. According to the procedures of Macrogen Japan Corp., the targeted fragments were amplified with thermal cyclers. Amplified PCR fragments were verified using agarose gel electrophoresis against known standards and then purified using the ExoSAP-IT Express PCR Product Cleanup Reagent (Thermo Fisher Scientific). Sequencing reactions were performed in a Bio-Rad C1000 or S1000 thermal cycler using the BigDye Terminator v3.1 Cycle Sequencing Kit (Thermo Fisher Scientific), according to the manufacturer’s instructions. Single-pass sequencing was performed on each template using 518F (5′-CCA GCA GCC GCG GTA ATA CG-3′) or 800R (5′-TAC CAG GGT ATC TAA TCC-3′) primers. Fluorescent-labeled fragments were purified from the unincorporated terminators either by ethanol precipitation or using the BigDye XTerminator Purification Kit (Thermo Fisher Scientific). Samples were analyzed using a 3730xl DNA Analyzer (Thermo Fisher Scientific).

The resulting sequences were assembled using MEGAX v10.1.7 (Tamura *et al.*, 2011). A single consensus sequence (1,473 bp) of the strain was elucidated. The 5′ and 3′ ends‍ ‍were manually aligned to truncate and refine both ends. The sequences of *Saccharospirillum* species and closely related species used in this study were obtained from the NCBI database (Fig. 1). Sequences were aligned using the CLUSTAL_W algorithm (Thompson *et al.*, 1997). Open and extended gap penalties were set at 10.0 and 5.0, respectively, for both the pair-wise and multiple alignment phases. Divergent rates in the completed alignments among *Saccharospirillum* species and strains were estimated using simple uncorrected pair-wise distance (p distance) matrices in MEGAX v10.1.7 (Tamura *et al.*, 2011).

Maximum-likelihood (ML) ana­lyses were conducted with 1,000 bootstrap replications using MEGAX ver. 10.1.7. The best-fit model for ML was obtained with partial (95%) deletion; no significant differences were noted in the best-fit model or phylogeny between the partial deletion and complete deletion approaches. In ML ana­lyses, the Kimura 2-parameter and gamma distribution (G+I) model were used for 16S rRNA gene regions.

Posterior probabilities for the Bayesian inference (BI) were calculated using MrBayes 3.1.2 (Huelsenbeck and Ronquist, 2001; Ronquist and Huelsenbeck, 2003) and the posterior probability distribution was estimated using the Metropolis-Coupled Markov Chain Monte Carlo (MCMCMC) method. MCMCMC from a random starting tree was used in this ana­lysis with two independent runs, one cold chain, and three heated chains with the temperature set at 0.2. Trees were sampled every hundredth generation for more than one million generations. To increase the probability of chain convergence, more than 500 trees were sampled after the standard deviations of the two runs decreased to <0.01 to calculate posterior probability. The number of burn-ins was 500.

Bacterial cells were fixed with glutaraldehyde (1% [v/v], final concentration) for a morphological ana­lysis. Fixed cells were collected on a 0.05-μm filter (111103 Whatman nuclepore track-etched membranes; Cytiva). The resultant filter was air-dried and mounted on scanning electron microscopy (SEM) specimen stubs, which were coated with osmium (thickness of 10‍ ‍nm) using an osmium coater (Osmium Plasma Coater OPC60A; Filgen). Cell images were obtained using SEM (SU1510; Hitachi).

In the whole-mount ana­lysis, 5‍ ‍μL of the fixed cell suspension was dropped onto a formvar-coated copper grid. After 5‍ ‍min, excess liquid was removed with filter paper, and a drop of 15 to 30× diluted EM stainer (Nisshin EM) was placed on the grid for 5‍ ‍s and then removed. After air drying, the specimen was observed using transmission electron microscopy (TEM) (HT7700; Hitachi).

The width and length of individual bacterial cells were calculated based on measurements from TEM images. According to equation (1) shown in Wang *et al.* (2008), the cell volume of bacterial cells was calculated as follows:

$$Cell\;volume=\frac{4}{3}\pi r^{3}+\pi r^{2}\left(L-2r\right)$$,

 (1)

where *r* represents half of the smallest width and *L* represents the length of the bacterial cell.

The effect of the filtration volume on bacterial passage through a 0.1-μm filter was exami­ned. Before and after filtration, the numbers of bacterial living cells were measured using the most probable number (MPN) method. Two bacterial strains, *Phaeobacter* sp. URN3 (Yamaguchi *et al.*, 2016a) and *Saccharospirillum* sp. SmNOM1 (present study), were used as controls. The former had no ability to pass through 0.2-μm or 0.1-μm filters, whereas the latter isolated from the *K. papilionacea* culture passed through 0.2-μm filters, but not 0.1-μm filters. As these strains proliferated in liquid media, the cultures became white and cloudy. Cloudiness before and after filtration was assessed.

Bacterial cultures that grew well in liquid media were diluted (10×) using fresh media. After an incubation for 1 h, 10, 20, and 40‍ ‍mL of the cell suspensions were filtered through 0.1-μm filters. In the cases of URN3 and SmNOM1, 20‍ ‍mL of the cell suspensions were used. The 0.1-μm filters were the track-etched, nucleopore, and polycarbonate membrane types (Whatman 111105; Cytiva). With a hand pump (MV8510; Mityvac), filtration was conducted under a pressure of less than 10 inHg (254‍ ‍mmHg).

Filtrates were serially diluted at 10^–1^–10^–11^ as described above, and then cultured in quintuplicate in 48-well clear plates (Iwaki) under dark conditions at 20°C for 5 d. Unfiltered cell suspensions (whole culture) were used as the control. The number of positive wells in which bacterial growth appeared was counted. Combinations of positive and negative wells were used to construct a statistical table. These procedures were used to calculate MPN.

In the present study, the marine bacterial strain, SspURN76, which is capable of passing through 0.1-μm filters, was isolated. Prior to inoculation into the sterile medium, the sediment suspension (original sample) was filtered through 0.1-μm filters. Liquid cultures showed a slightly white and cloudy color, and bacterial colonies appeared on the black-stained agar plate. In contrast, bacterial cultures of *Phaeobacter* sp. URN3 and *Saccharospirillum* sp. SmNOM1 did not proliferate under the same filtration procedures. Notably, SmNOM1 cultures grew well when filtered through 0.2-μm filters.

SspURN76, along with SmNOM1, belonged to the genus *Saccharospirillum* (*Gammaproteobacteria*) in the mole­cular phylogenetic tree (Fig. 1). Partial 16S rRNA gene sequences of SspURN76 were matched by >98% with those of *Saccharospirillum correiae* and *Saccharospirillum mangrovi*; the p distance was closer to the former (0.00987) than to the latter (0.01802) among *Saccharospirillum* strains (Table S1). SspURN76 was genetically different (p distance: 0.01652), with SmNOM1 being incapable of passing through the 0.1-μm filter (Table S1). Additionally, the p distance was close (0.00450) between *Saccharospirillum alexandrii* and *Saccharospirillum impatiens*.

TEM images displayed slender cells that possessed a long flagellum (Fig. 2). Cell width and length were 0.09–0.19 and 2.16–4.70‍ ‍μm, respectively (*n*=30, Figs. 2 and 3). The minimum cell size of SspURN76 was 0.09×3.2‍ ‍μm (Fig. 3). *Saccharospirillum* species, except for *S. salsuginis*, are spirillum-shaped bacteria. Among *Saccharospirillum* species, SspURN76 cells showed a similar filamentous slender shape to that of *S. mangrovi* cells (Fig. 3). A comparison of minimum cell widths between *S. mangrovi* (0.3‍ ‍μm) and SspURN76 (0.09‍ ‍μm) confirmed that SspURN76 cells were slender. Assuming the slender and spirillum-like cell shape, the cell volume of SspURN76 was estimated to be 0.022–0.095‍ ‍μm^3^ (*n*=30). *Saccharospirillum* sp. SspURN76‍ ‍ap­peared to be an ultra-slender marine bacterium with a cell volume <0.1‍ ‍μm^3^.

We herein describe the marine bacterial strain, *Saccharospirillum* sp. SspURN76, which is capable of passing through a 0.1-μm filter. The cells of both *Saccharospirillum* sp. SspURN76 (present study) and *H. gracilis* (Wang *et al.*, 2008) showed a slender filamentous shape. As reported by Nakai (2020), *Silvanigrella paludirubra* and *Fluviispira multicolorata* recently described by Pitt *et al.* (2020) are in a category of slender filamentous bacteria and appear to be rod- and/or filamentous-shaped bacteria with widths >0.2‍ ‍μm (see Fig. 1 in Pitt *et al.*, 2020). To the best of our knowledge, it has not yet been established whether *S. paludirubra* and *F. multicolorata* are capable of passing through 0.1-μm filters. We consider a bacterial slender shape similar to a hair to be decisive in the passage of bacteria through filters. Wang *et al.* (2008) showed that *H. gracilis* cells had not only the smallest width of 0.2‍ ‍μm, but also a slender shape. Furthermore, SspURN76 cells were slender with a minimum width of 0.09‍ ‍μm. These findings suggest that the ultra-slender shape of SspURN76 is a critical factor for its passage through a 0.1-μm filter.

The MPN of the tested SspURN76 cultures was 0.98×10^8^‍ ‍mL^–1^, which is close to the density of living cells (Fig. 4). Most, but not all, living cells passed through 0.1-‍μm filters depending on the filtration volume, and 28% of all cells passed through these filter when 40‍ ‍mL of culture was filtered (Fig. 4). The filtration volume affected the passage of *Saccharospirillum* sp. SspURN76 through the 0.1-μm nuclepore filter (the present study), which is consistent with previous findings on *H. gracilis* (Wang *et al.*, 2008). This phenomenon may be attributed to the change in the bacterial concentration directly above the filter surface (Wang *et al.*, 2008). Cell concentrations above the filter surface increase when retaining bacterial cells and increasing the filtration volume; bacterial cells may then be pushed and/or push other cells into micropores.

Bacterial cell sizes and their passage through filters with micropores vary. Some field populations in coastal waters initially pass through 0.2-μm filters, but subsequently lose this passage in nutrient-enriched media due to cell enlargement (Obayashi and Suzuki, 2019). Torrella and Morita (1981) showed an increase in the size of freshly incubated bacterial cells. This study used culture media that allowed *Saccharospirillum* sp. SspURN76 and other species (Urata *et al.*, 2022) to proliferate well. SspURN76 cells in the media clearly exhibited a nutrient-repleted state and were able to pass through 0.1-μm filters (Fig. 4). The present results strongly suggest that the passage of SspURN76 through a 0.1-μm filter is not transient, but may vary among individual cells.

Bacterial passage through micropores of filters may be affected by cell states and filtration procedures. Cell shape, size, flexibility, and elasticity as well as the vacuum pressure and sample volume at the filtration procedure are factors that may affect bacterial passage. Therefore, comparatively examining the morphological and physiological states of SspURN76 cells in various filtration procedures will be necessary in the future to understand bacterial passage through micropore filters.

To remove bacterial cells and prepare extremely small particles, such as diatom viruses (size 0.02–0.04‍ ‍μm), from aquatic environments, our laboratory occasionally freezes sediment suspensions and/or water samples, filtrates the thawed samples through 0.2-μm and 0.1-μm filters, and then inoculates the filtrates into axenic diatom cultures. In our experience, cultures rarely become cloudy during incubations. To obtain bacterial cells from such a fraction, we inoculated the sediment sample into algal cultures and isolated *Saccharospirillum* sp. SspURN76. No algicidal activity of the bacterium culture was detected during these procedures. The freezing of samples and culturing them with diatom cells may affect the isolation of SspURN76.

Marine bacteria, such as *Saccharospirillum* sp. SspURN76, may contribute to filtration sterilization failure. Cells of *Saccharospirillum* species have widths >0.3‍ ‍μm (Fig. 3) and, thus, are unlikely to pass through filters with micropores. Nakai *et al.* (2013) isolated *Saccharospirillum* kure and NOW (Fig. 1 and Table S1) from 0.2-μm filtered seawaters collected at Hiroshima and Okinawa, respectively. Furthermore, the present study showed that SmNOM1 passing through a 0.2-μm filter was closely related to *Saccharospirillum* kure (Fig. 1). Therefore, some *Saccharospirillum* species appear to pass through filters with 0.2-μm and/or 0.1-μm pores and may contribute to filtration sterilization failure. However, these failures are infrequent. Previous studies, particularly those that employed sterilization with various chemicals and seawater samples, reported that 0.2-μm and/or 0.1-μm filtration procedures were effective. Specifically, filtration procedures using 0.1-μm filters ensured sterilization in most cases.

Marine bacterial cells trapped on 0.2-μm filters, and even on 0.1-μm filters, are unlikely to represent the full spectrum of microorganisms present. To obtain a comprehensive understanding of microbes in marine environments, future studies that focus on monitoring marine bacteria in not only 0.2-μm, but also 0.1-μm filtrates are needed. The present results indicate the potential of *Saccharospirillum* sp. SspURN76 as a standard for 0.1-μm pore filterable bacteria.

## Citation

Yamaguchi, H., and Yamada, K. (2025) Characterization of a Marine Bacterium Passing through a 0.1-μm Pore-sized Filter. *Microbes Environ ***40**: ME24014.

https://doi.org/10.1264/jsme2.ME24014

## Supplementary Material

Supplementary Material

## Figures and Tables

**Fig. 1.**
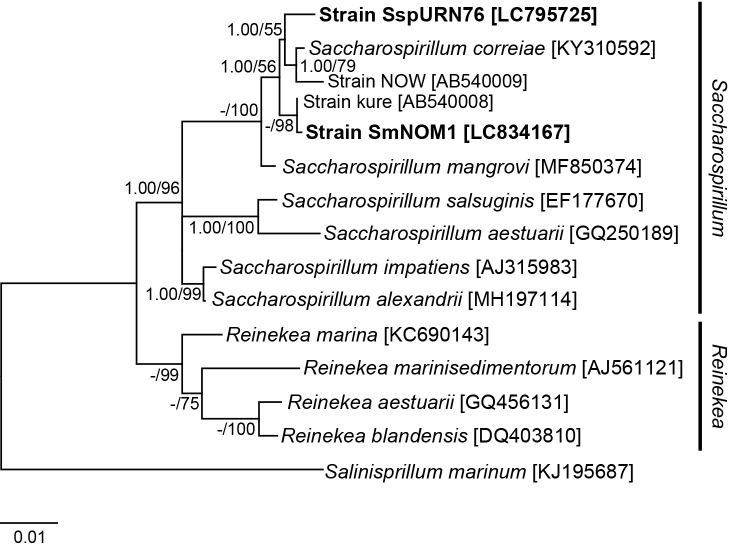
Maximum likelihood phylogenetic ana­lysis of *Saccharospirillum* and other genera based on 16S rRNA gene sequences. Bayesian posterior probability values are shown with bootstrap percentage values (*n*=1,000) from a maximum-likelihood ana­lysis at each branching position; a hyphen indicates that branching was not supported in Bayesian ana­lyses. The strains isolated in the present study and their accession numbers are shown in bold font. *Reineke* and *Salinispirillum* species are used as the outgroup genera close to *Saccharospirillum*.

**Fig. 2.**
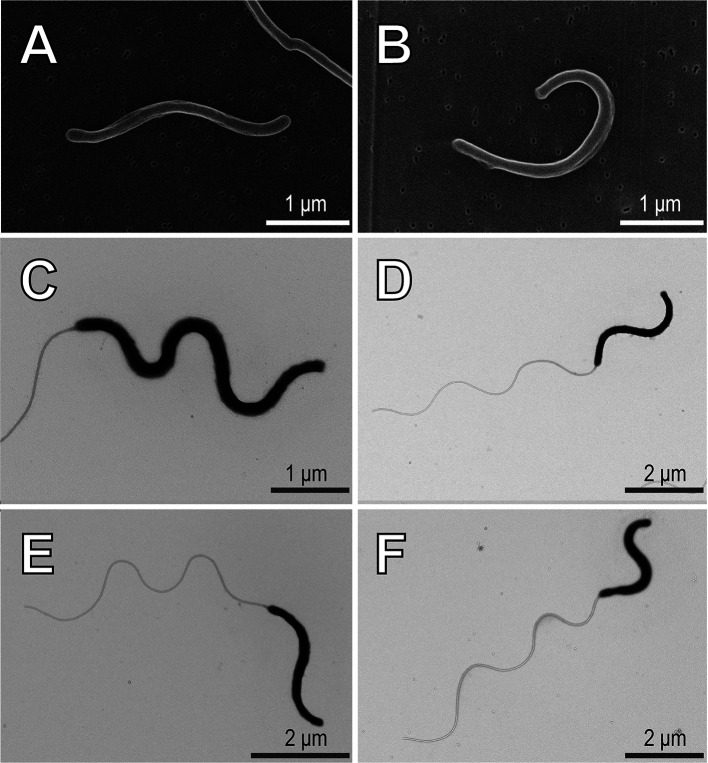
Cell images of *Saccharospirillum* sp. SspURN76 cultures shown by scanning electron micrographs (A and B) and whole-mount transmission electron micrographs (C, D, E, and F).

**Fig. 3.**
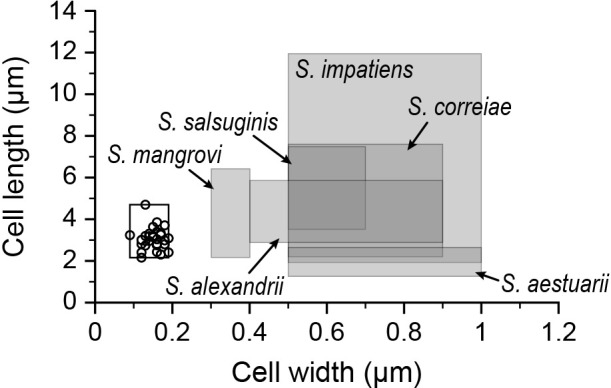
Cell width and length of *Saccharospirillum* species (shaded square areas), including SspURN76 (white square area). Circles indicate cell length and the smallest width of individual cells of SspURN76 (*n*=30). Size data are referred to as follows: *S. aestuarii* (Choi *et al.*, 2011), *S. alexander* (Yang *et al.*, 2020), *S. correiae* (Fidalgo *et al.*, 2017), *S. impatiens* (Labrenz *et al.*, 2003), *S. mangrovi* (Zhang *et al.*, 2018), and *S. salsuginis* (Chen *et al.*, 2009).

**Fig. 4.**
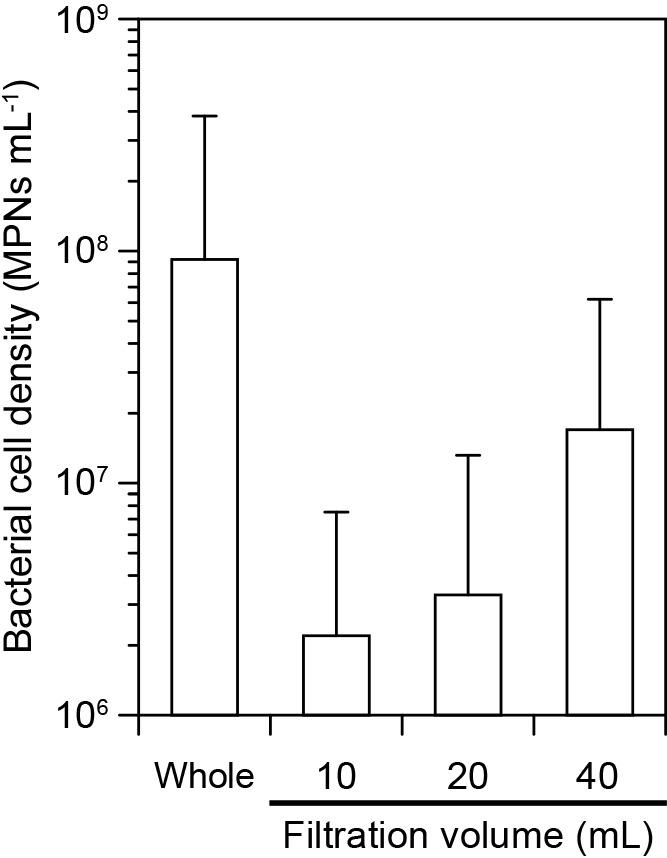
Living-cell densities of *Saccharospirillum* sp. SspURN76 cultures diluted (10×) using fresh media, incubated for 1 h, and filtrated through a 0.1-μm pore-sized filter. A serial dilution method (quintuplicate) calculated living cells as the most probable number (MPN). Error bars show 95% confidence intervals.
