# Supplementary material for: Characterization of a Marine Bacterium Passing through a 0.1-μm Pore-sized Filter
Source: Microbes Environ. 2025 Mar 8;40(1):ME24014. doi: 10.1264/jsme2.ME24014 (PMC11946411; doi:10.1264/jsme2.ME24014)
Supplement: Supplementary file 1 — Supplementary Material [file 40_24014_s1.pdf]

Table S1 Uncorrected p-distance values of partial 16S rRNA gene sequences among *Saccharospirillum* species/strains.

Bacteria and Accession number	SspURN76 LC795725	SmNOM1 LC834167	*Saccharosprillum* kure AB540008	*Saccharosprillum* NOW AB540009	*S. salsuginis* EF177670	*S. aestuarii* GQ250189	*S. impatiens* AJ315983	*S. mangrovi* MF850374	*S. alexandrii* MH197114	*S. correiae* KY310592
SspURN76 LC795725										
SmNOM1 LC834167	0.01652									
Saccharosprillum kure AB540008	0.01502	0.00150								
Saccharosprillum NOW AB540009	0.02104	0.01953	0.01953							
S. salsuginis EF177670	0.03681	0.04132	0.04132	0.04887						
S. aestuarii GQ250189	0.04586	0.04586	0.04586	0.05719	0.02331					
S. impatiens AJ315983	0.04580	0.03453	0.03604	0.04207	0.02928	0.03681				
S. mangrove MF850374	0.01802	0.01351	0.01502	0.01653	0.04358	0.05188	0.03453			
S. alexandrii MH197114	0.04279	0.03303	0.03453	0.03907	0.03228	0.03982	0.00450	0.03153		
S. correiae KY310592	0.00987	0.01367	0.01215	0.01064	0.03721	0.04635	0.03338	0.01519	0.03035	

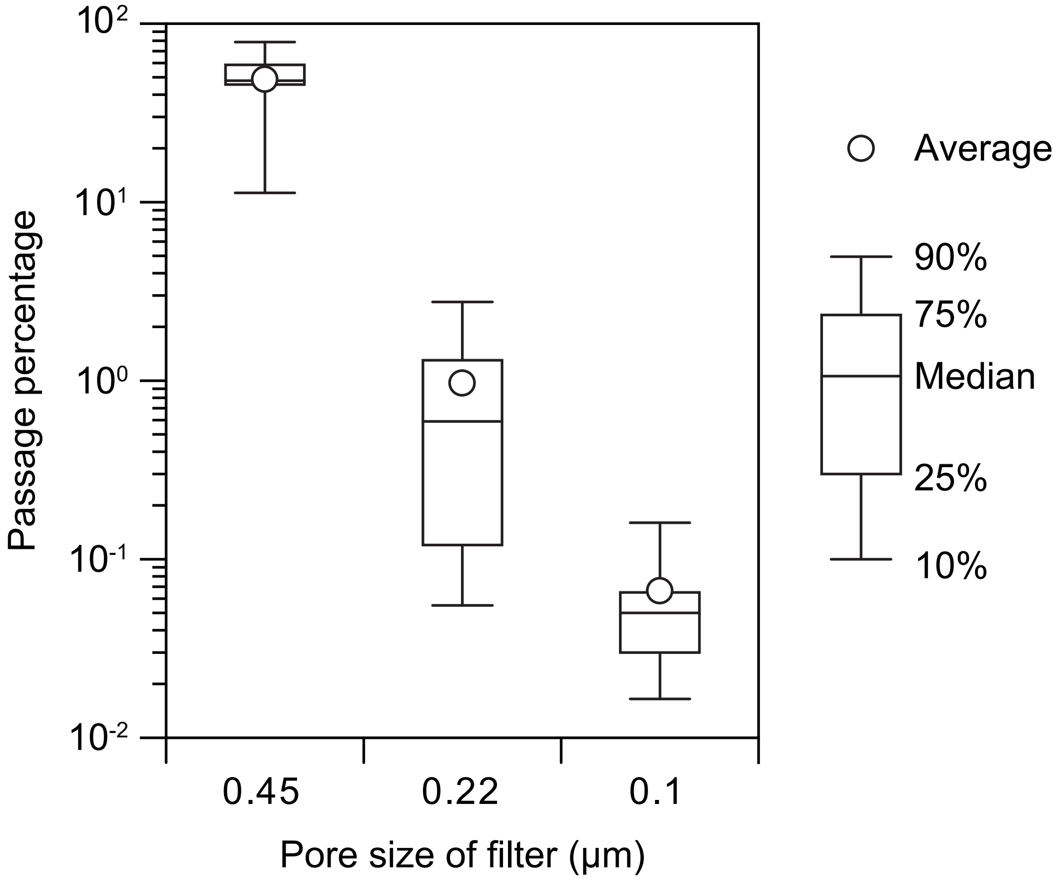

Fig. S1 Re-summarized plots of Table 1 shown in Wang *et al*. (2007). The box-and-whisker plots indicate the passage percentage of bacterial cells in freshwater samples (n=10) through pre-sterilized commercially available syringe filters (0.1, 0.22, and 0.45 μm pore size). The top and bottom bars indicate upper 10% and lower 90% values, respectively. Boxes indicate 25% and 75% values. The bars and circles in the boxes are the median and averaged values, respectively.
